# Combined Orthodontic and Restorative Minimally Invasive Approach to Diastema and Morphology Management in the Esthetic Area. Clinical Multidisciplinary Case Report with 3-Year Follow-Up

**DOI:** 10.1155/2020/3628467

**Published:** 2020-06-09

**Authors:** L. Giannetti, R. Apponi

**Affiliations:** Department of Surgical, Medical, Dental and Morphological Sciences with Transplant, Oncological and Regenerative Medicine Interest, University of Modena and Reggio Emilia, Modena, Italy

## Abstract

**Introduction:**

Ceramic laminates are restorations that are bonded using adhesive techniques, which provide for the treatment of the prepared dental elements according to well-defined steps. Adhesive cementation guarantees high predictability and esthetics. Orthodontic treatment is the first choice in patients with a dental misalignment. Patients who have dental element color and shape issues will undergo restorative treatment following orthodontics. *Case Report*. This clinical report describes a case treated with an interdisciplinary approach (orthodontic and prosthetic) of a patient who presented with diastemas, inversion of dental axes, small clinical crowns, and chromatic imperfections in the anterior maxillary teeth. The esthetic expectations of the patient for the anterior portion of the maxillary anterior teeth have been successfully achieved through orthodontic treatment and the realization of ceramic veneers. An accurate interdisciplinary evaluation of the treatment was necessary for a satisfactory result in the anterior maxillary teeth esthetically compromised in several aspects. *Discussion*. The modern materials used with the strict protocol of the adhesive procedures allow a minimally invasive, highly esthetic approach with an excellent long-term prognosis. The restorations must reproduce the physiological characteristics of the natural teeth aiming at an excellent biological, biomechanical, functional, and esthetic integration. Many adult patients come to visit with a combination of problems; the esthetic rehabilitation of these cases requires the evaluation of the quantity of gingival exposure, of the gingival architecture, of the size of the clinical crowns, and of the dental position. The ideal treatment of these cases involves an interdisciplinary approach. Prosthodontists, periodontists, orthodontists, and dental technicians must work together because the understanding of the various phases of treatment is fundamental to achieve the desired clinical result.

**Conclusion:**

The coordinated treatment of the orthodontist, periodontist, and prosthodontist, with careful consideration of the expectations and requests of patients, was fundamental for the success and satisfaction of the patient.

## 1. Introduction

Ceramic veneers are a highly conservative treatment compared to total crowns as they require minimally preparation [1]. The preparation for ceramic veneers should be limited to enamel even if the exposure of dentin areas is inevitable, especially in the cervical areas, as discussed by Chai et al. [2]

The treatment with ceramic veneers adhesively cemented allows to obtain improvements in color, shape, positioning, reestablishment of the vertical dimension of the occlusion, and teeth exposure [3]. According to Aboushelib et al. [4], “Once cemented correctly, ceramic veneers become an integral part of the structure of the tooth and share part of the loading stresses applied during the chewing cycle.”

Patients with significant dental misalignment should have orthodontic movement as their first choice of treatment as a more conservative option. However, those who have defects in the color or shape of teeth will already be subject to a more invasive restorative treatment for cosmetic correction. In other cases, restorative and orthodontic therapies can be combined to obtain a better result and to guarantee the most conservative possible approach. In cases like this presented, the orthodontic alignment through the use of aligners has allowed the repositioning of the dental elements in a strategic way, in order to make the preparation minimally invasive by limiting it to the enamel to obtain an ideal adhesive bonding.

The aim of this article is to present an illustrative clinical case with an accurate operative protocol for the esthetic rehabilitation of a patient through the combination of orthodontic therapy and prosthetic therapy.

## 2. Case Report

A 54-year-old female patient came to dental office to improve her smile. There were no relevant medical history and no contraindication to dental treatment.

The extraoral examination indicated a symmetrical and mesognathic facial pattern with a convex profile. At rest, there were about 2 mm of incisal edge visible with slightly incompetent lips.

**Figure 1:**
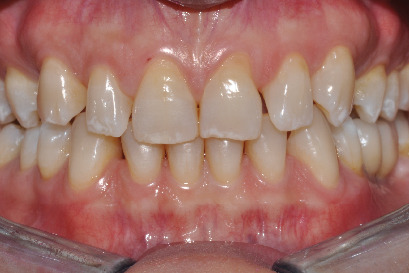
Initial intraoral situation.

On the intraoral objective examination, the patient presented an all-ceramic crown on the element 16, two crowns screwed onto implants at the sites of the elements 35 and 36, and Miller's I class gingival recessions on the teeth 11, 21, 37, 34, and 45. The patient's biotype was thick, and there were no periodontal problems (Figure 1). The movements of laterality and protrusion had no working and nonworking precontacts. The overbite was 3.5 mm and the overjet 1 mm.

The patient presented a canine and molar 1st dental class. The lower midline coincided with the facial median while the upper midline was displaced 2 mm to the right. The smile line was medium, the incisal trend compared to the lower lip was convex, the lip was 1 mm apart, the dental exposure was 8 teeth (from 14 to 24), and the labial corridor was normal both on the right and on the left.

From the esthetic analysis of the profile, an adequate support of the upper lip was observed for the correct inclination of the upper incisors. At the dental esthetic analysis, the inversion of the dental axis of teeth 12 and 22 was found. Problems were present at the level of the gingival margins and at the embrasures lines. There were diastemas between all maxillary teeth with the exception of the space between 11 and 21.

A surface of wear was found on the incisal edge of tooth 21, and there was the presence of white spots on 12, 11, 21, 22, and 23.

Subsequently, posterior radiographs were performed to assess the presence of interproximal carious lesions, photographs were taken, and dental impressions were taken in order to make a diagnostic wax-up. At the time of the reevaluation, the presence of carious and periodontal lesions was not found. The diagnosis included the presence of diastemas between the upper front teeth, the incorrect position of the elements 12 and 22, the presence of white spots, the nonideal proportion of the elements 11 and 21, and the noncoincidence of the facial midline with the upper dental midline.

The treatment of the white spots could have been performed using infiltrating resin (Icon, DMG) resin with a superficial [5] or deep [6] treatment. However, the patient wanted a change not only in the color and surface texture of his teeth but also in morphology.

It was decided to treat the upper anterior teeth with a minimally invasive multidisciplinary orthodontic-prosthetic approach. The proposed treatment plan was to move the upper teeth to redistribute the diastemas between 14 and 24, restore a correct surface texture, and restore the position of the upper dental midline and the proportion of the teeth using feldspathic ceramic veneers.

**Figure 2:**
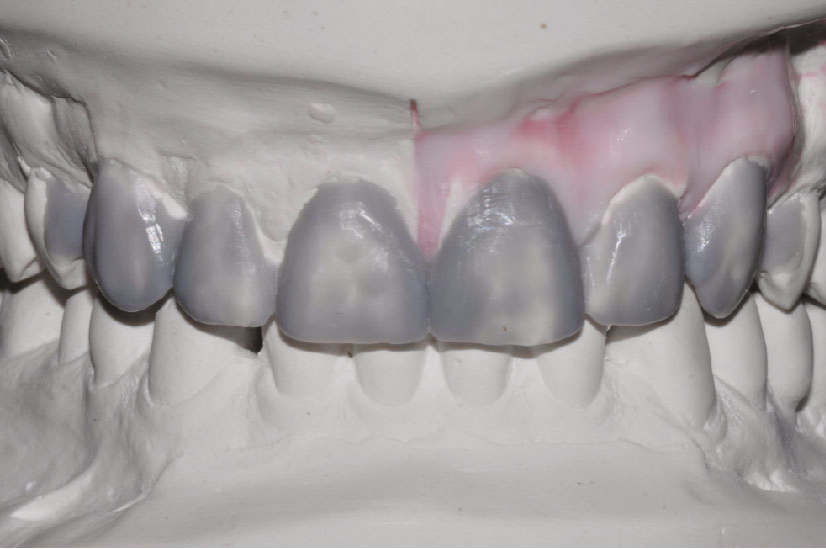
Initial diagnostic wax-up.

On the study models originated by preliminary impressions, the technician performed an initial wax-up (Figure 2) on which he fabricated a silicone template (Vestige 70 shore, Trayart) to print the preliminary mock-up in the patient's mouth.

**Figure 3:**
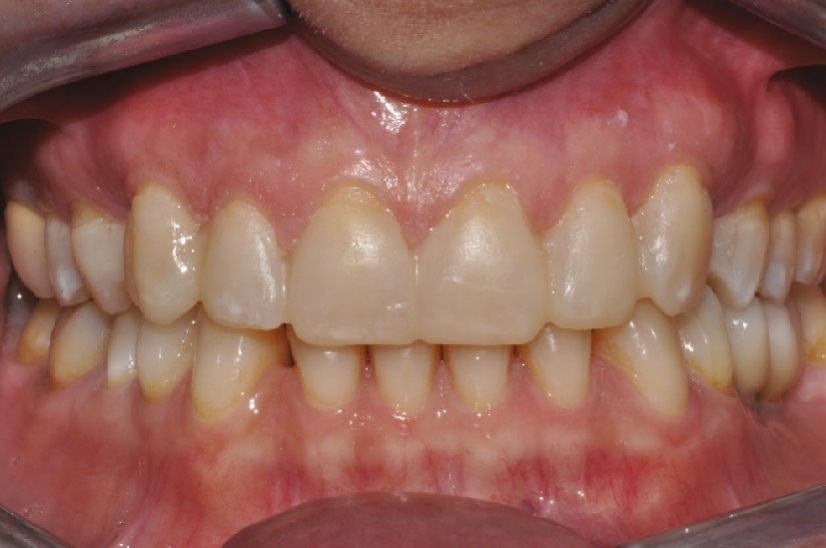
Initial mock-up.

The flowable composite resin is then dispensed in to the silicone template and positioned in the patient mouth (Figure 3). The previsualization provided a true copy of the planned wax-up allowing for an immediate evaluation of shape, volume, occlusion, and relationship with the surrounding tissue. The patient thus accepted the treatment plan. Impressions in polyvinylsiloxane (Aquasil, Dentsply) were taken, and a teleradiography and an orthopantomography were performed. X-rays and impressions were sent to the Invisalign® (Align Technology) center together with the orthodontic treatment goals.

The cephalometric radiography study has allowed to appreciate the following: severely brachifacial growth type; 1st skeletal and dental class; poor sagittal development of the maxilla; and increased mandibular development.

**Figure 4:**
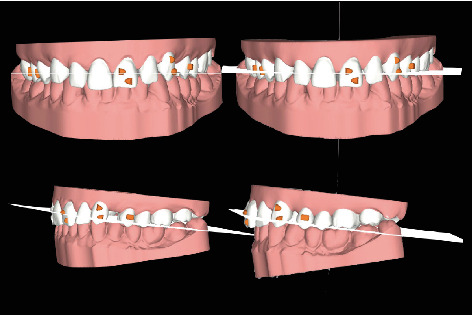
Orthodontic treatment plan with the Invisalign® software. On the left, the initial situation (frontal and lateral view) and, on the right, the case at the end of the orthodontic treatment.

The orthodontic treatment of the upper arch alone was carried out using transparent aligners (Figure 4), in order to redistribute the diastemas present in the superior frontal group so as to uniform the spaces, center the medians, and facilitate the subsequent prosthetic rehabilitation of the same. In addition to aligning the upper frontal group from 13 to 23, derotation of the 23 and correction of the distal tipping were performed.

**Figure 5:**
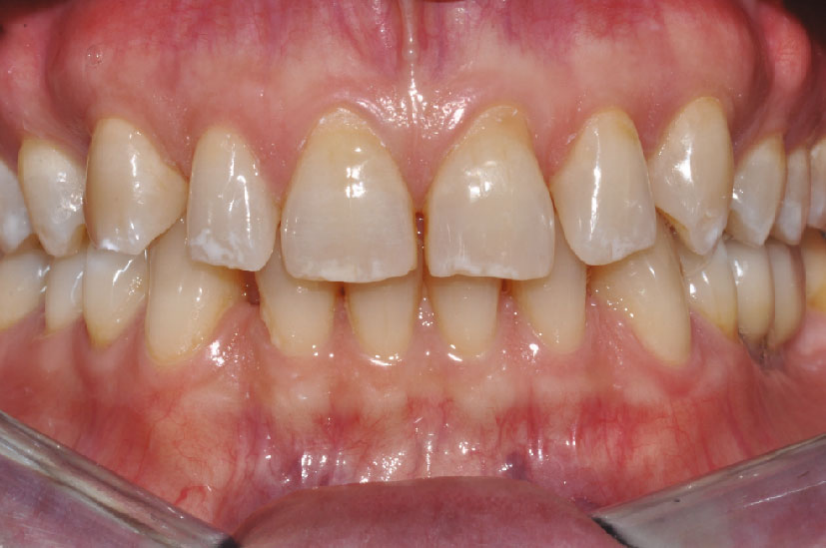
Completion of orthodontic treatment.

**Figure 6:**
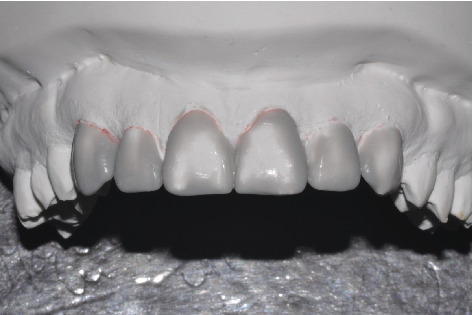
Final wax-up.

**Figure 7:**
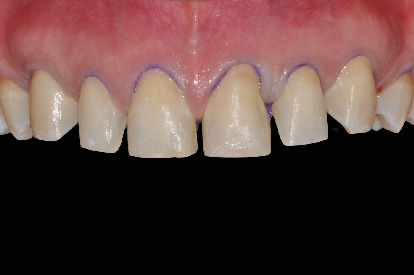
Final teeth preparation.

**Figure 8:**
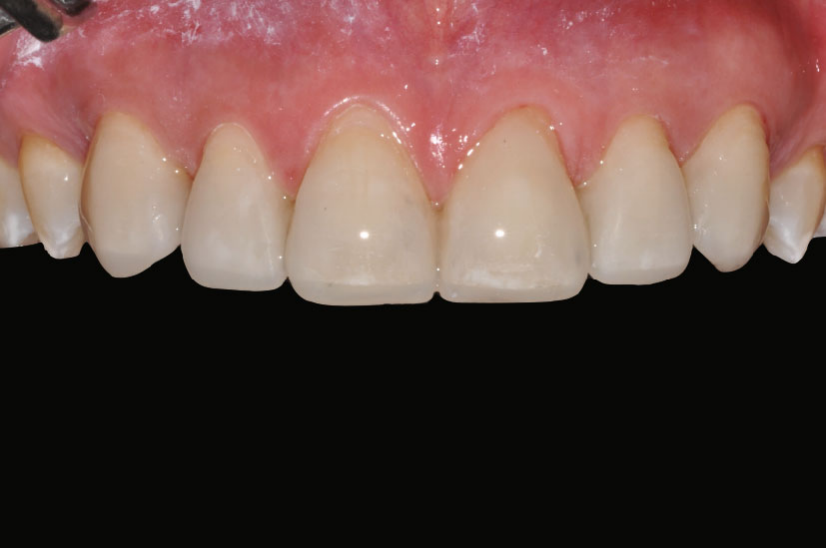
Mock-up printing on final preparations.

After six months, the orthodontic treatment was completed (Figure 5), alginate impressions of the dental situation with the redistributed diastemas were taken. The technician developed class IV plaster models and made the final wax-up on them (Figure 6), making a silicone template for the definitive mock-up. The mock-up was printed in the patient mouth after performing computerized anesthesia. The preparation of teeth 13, 12, 11, 21, 22, and 23 was performed through the mock-up so that they could be minimally invasive and preserve the greatest possible amount of enamel (Figure 7) [7]. In the mesial parts of the elements 14 and 24, two direct composite resin restorations were performed. Once the teeth were prepared, the retractor fibers (Retraction Cord #00, Ultrapak™) were positioned, and an impression in polyether (Impregum Penta, 3M ESPE) was taken. The mock-up was printed again on the prepared teeth, and it was finished and left in the patient's mouth as a temporary (Figure 8). The dental technician received the impressions, developed the model, and stratified sintered feldspathic veneers from tooth 13 to tooth 23.

After 7 days, ceramic veneers were made, and the patient returned at the dental office. The feldspathic ceramic veneers were etched with 9% hydrofluoric acid from 60 to 90 seconds, rinsed, and then cleaned with pure alcohol for 5 minutes. The silane was placed on them, and then the adhesive was brushed on without light curing. The prepared teeth were etched with 35% orthophosphoric acid for 30 seconds and rinsed for 30 seconds, and then the primer and the bonding were applied on without light curing. Resinous composite cement (Variolink Esthetic, Ivoclar Vivadent) was applied, the veneers were positioned on the teeth, and the cement excesses were removed.

Each veneer was light-cured for 120 seconds.

**Figure 9:**
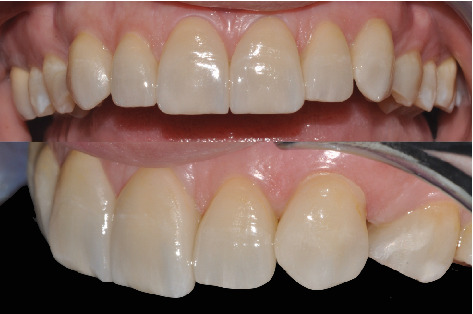
Final result with 3-year follow-up.

Finishing was carried out with fine-grain diamond burs and decreasing abrasiveness rubber burs. The patient returned at the office every six months for maintenance calls, and 3 years after the cementation, the control photographs and radiographs were taken (Figure 9).

## 3. Discussion

The growing demand for esthetic restorations [8] can be satisfied with the currently available ceramic materials, since the absence of metal allows the transmission of light through the restoration and allows a chromatic correspondence with the natural dentition [9].

Adhesive dentistry allows respect for the esthetics, function, and preservation of healthy dental tissue [10]. The modern ceramic and composite materials used with the strict protocol of the adhesive procedures allow a minimally invasive, highly esthetic approach with an excellent long-term prognosis [11]. The restorations must reproduce the physiological characteristics of the natural tooth aiming at an excellent biological, biomechanical, functional, and esthetic integration [9, 12, 13].

The esthetic rehabilitation by adhesive procedures can be performed with direct composite restorations, feldspathic ceramic, composite veneers, lithium disilicate, or zirconia crowns. Feldspathic ceramic and lithium disilicate are today the most-used ceramics for their esthetic qualities. Furthermore, the cohesion between conditioned veneers with hydrofluoric and silane and the composite determine excellent adhesion with the treated enamel [14].

According to the Magne and Belser [15] classification that describes three main indications for an indirect rehabilitative approach in an esthetic area, the case presented in this article is a type IIB, a case that needs major morphological changes to close internal diastemas and triangles.

The innovative design of current preparations for ceramic veneers is much less invasive than the design of traditional full crown preparations [16]. Edelhoff and Sorensen [17] quantified, with a gravimetric analysis, the amount of dental structure removed with a modern type of preparation: ceramic veneers require a less extensive preparation from a quarter to half compared to complete crown preparations.

The success of an esthetic treatment with veneers derives from a correct planning and from the accuracy in the execution of every single step of the treatment [14].

Many adult patients show up with a combination of problems; the esthetic rehabilitation of these cases requires the evaluation of the quantity of gingival exposure, of the gingival architecture, of the size of the clinical crowns, and of the dental position [18–20]. The ideal treatment of these cases involves an interdisciplinary approach.

Prosthodontists, periodontists, orthodontists, and dental technicians must work together because the understanding of the various phases of treatment is fundamental to achieve the desired clinical result [14].

Orthodontic treatment in adult patients can be matched to the prosthetic and surgical treatments in order to reduce the invasiveness and to improve the esthetics and function of the final result [21]. The interproximal diastemas can be strategically redistributed, and the teeth can be positioned to reduce the thickness of the preparations and restorative materials [22].

The previewing of the final result can be a key motivation, not only to start the treatment but also to keep the patient involved throughout the process [23].

In the present case, the patient's esthetic needs were met through an interdisciplinary treatment approach consisting of orthodontic movements, temporary restorations, and a combination of porcelain veneers and direct composite restorations.

## 4. Conclusion

This clinical report describes an interdisciplinary approach in which communication and coordination have been fundamental for a better esthetic result in the anterior jaw. The coordinated treatment of the orthodontist, periodontist, and prosthodontist, with careful consideration of the expectations and requests of patients, was fundamental for success and patient satisfaction.
